# Neural signatures of serial order access in verbal working memory

**DOI:** 10.1093/cercor/bhag160

**Published:** 2026-10-12

**Authors:** Giulia Cristoforetti, Yangwen Xu, Simone Viganò, Jean-Philippe van Dijck, Roberto Bottini, Wim Fias

**Affiliations:** Center for Brain and Mind, University of Trento, Piazza Manifattura 1, 38068, Rovereto, Italy; Department of Experimental Psychology, Ghent University, Henri Dunantlaan 2, 9000 Ghent, Belgium; Max Planck Institute for Human Cognitive and Brain Sciences, Stephanstraße 1a, 04103 Leipzig, Germany; EA/DRF/Inst. Joliot, NeuroSpin, INSERM, Cognitive Neuroimaging Unit, Université Paris-Saclay, Bâtiment 145 - Point Courrier 156, Gif/Yvette, 91191, France; Department of Experimental Psychology, Ghent University, Henri Dunantlaan 2, 9000 Ghent, Belgium; Department of Applied Psychology, Expertise Centre for Care and Welfare, Thomas More University of Applied Sciences, Campus Sanderus, Molenstraat 8, Antwerp, 2018, Belgium; Center for Brain and Mind, University of Trento, Piazza Manifattura 1, 38068, Rovereto, Italy; Department of Experimental Psychology, Ghent University, Henri Dunantlaan 2, 9000 Ghent, Belgium

**Keywords:** fMRI, multivoxel pattern analysis, ordinal position, parietal cortex, spatial attention

## Abstract

Serial order working memory allows information to be maintained as structured sequences, yet the neural formats supporting access to serial position remain unclear. Behavioral evidence suggests that temporal order can be recoded into spatial representations, but serial order may also rely on representations that generalize beyond the spatial configuration of encoding. Here, participants memorized digit sequences presented either left-to-right or right-to-left, thereby reversing the mapping between ordinal position and external space while keeping retrieval cues centrally presented during fMRI. Behaviorally, spatial coding during retrieval was modulated by encoding direction. Multivoxel pattern analysis revealed direction-specific decoding in lateral parietal cortex: left inferior parietal cortex encoded serial position after left-to-right encoding, whereas right inferior parietal cortex did so after right-to-left encoding. In contrast, cross-direction decoding revealed direction-invariant effects in left supramarginal and precentral regions, suggesting serial-position information that generalized across spatial layouts. Finally, univariate parametric modulation revealed activity scaling with serial position in bilateral posterior parietal cortex, left anterior hippocampus, and action-control regions. Together, these findings indicate that serial order access in working memory relies on multiple neural signatures, including spatially context-dependent representations, direction-invariant ordinal codes, and graded process-related activity.

## Introduction

Serial order working memory (WM) enables the temporary maintenance of sequential information and supports higher cognitive functions such as language and action (Baddeley 2012). Remembering a sequence requires preserving not only item identity, but also the order in which items occurred. Because accessing a specific position often involves traversing an internal representational structure rather than passively retrieving stored items (Sternberg 1966; Marshuetz 2005), a longstanding question concerns how serial position is represented in the brain and which principles govern access to this information.

When information is presented sequentially, items are inherently embedded in a temporal context. Serial order processing does not appear to rely solely on direct inter-item associations, but on organizing items within an internal framework that allows positions to be marked and accessed (Henson 1998; Oberauer 2009). Recent accounts further suggest that WM requires mechanisms that bind item content to its spatiotemporal context, such as content-independent “pointers” (Awh and Vogel 2025). However, the nature of this framework, and how items are assigned to and accessed within it, remains unclear.

One account proposes that temporal order is translated into a spatially organized representational structure that can be accessed through spatial attention mechanisms (Bonato et al. 2012; Abrahamse et al. 2017). In left-to-right reading cultures, serial positions are typically organized along an internal horizontal axis, with early and late items associated with left and right space, respectively (van Dijck and Fias 2011), whereas the reverse pattern has been observed in right-to-left reading cultures (Rasoulzadeh et al. 2024). Access to a specific item may occur either by directly accessing its position or by serially scanning through ordered positions. Behavioral evidence for this view comes from the ordinal position effect (OPE), typically observed in probe-recognition tasks in which participants memorize a centrally presented sequence and respond to a property of a probe item using left or right response keys. In left-to-right reading cultures, early items elicit faster left-hand responses, whereas later items elicit faster right-hand responses (van Dijck and Fias 2011). Converging evidence shows that accessing early versus late items is accompanied by leftward and rightward shifts of spatial attention (van Dijck et al. 2013; De Belder et al. 2015; Rasoulzadeh et al. 2021) and by systematic eye movements during retrieval (Schroth et al. 2025). Together, these findings suggest that temporal order can be recoded into a spatial format that supports access to memory representations through spatial attention mechanisms (Abrahamse et al. 2014).

Importantly, spatial coding of serial order is flexible and can be shaped by the spatial layout present during encoding. In a probe-recognition task with sequences presented from right to left, left-to-right readers showed an association between early items and the right side and between later items and the left side, reversing the default left-to-right mapping typically observed for temporal order (Bonato et al. 2012; Guida et al. 2020). This suggests that serial order representations are not fixed, but can be shaped by the spatial characteristics of the encoding context.

This does not imply that temporal information becomes irrelevant. Evidence from position-cued retrieval tasks shows that temporal and spatial dimensions can make dissociable contributions to serial order processing in WM (Baddeley and Hitch 1974; Farrell et al. 2026). When successive items are presented at random spatial locations, sequence structure can be preserved even when spatial cues are inconsistent or reversed, indicating that order can be maintained beyond the specific spatial layout of encoding. Together, these findings suggest that serial order may rely on multiple representational formats that differ in their dependence on the encoding context (Majerus 2019). Here, we use “encoding context” to refer to the spatial configuration in which the sequence is experienced when serial positions are mapped onto distinct locations. Context-dependent representations are therefore defined as those preserving information about this configuration, whereas context-independent representations capture ordinal structure in a format that generalizes beyond the spatial layout.

Recent fMRI work shows that both parietal and medial temporal regions contribute to serial order retrieval in WM, with multivariate analyses revealing that serial position can be decoded from both the intraparietal sulcus (IPS) and the hippocampus (Cristoforetti et al. 2022). However, these findings do not specify whether these regions support the same representational format or different components of serial order coding.

One possibility is that parietal and medial temporal regions differentially support representations that vary in their dependence on the encoding context. Based on their established roles in attentional orienting and ordinal comparison, parietal regions, particularly the intraparietal sulcus (IPS), may preferentially express context-dependent codes shaped by the spatial configuration of encoding (Corbetta and Shulman 2002; Nobre et al. 2004; Vandenberghe and Gillebert 2009; Bressler and Silver 2010; Attout et al. 2014). In contrast, medial temporal regions, including the hippocampus, may support more general sequence-based representations that capture ordinal structure beyond the specific spatial layout, consistent with their role in relational memory and sequence coding across spatial and nonspatial domains (Jenkins and Ranganath 2010; Eichenbaum 2014; Tubridy and Davachi 2011; Constantinescu et al. 2016; Garvert et al. 2017; Behrens et al. 2018; Bellmund et al. 2018; Theves et al. 2019; Bellmund et al. 2020; Bottini and Doeller 2020).

This parietal–medial temporal distinction is unlikely to exhaust the neural architecture of verbal serial-order memory. Verbal order processing also recruits a predominantly left-lateralized frontoparietal network, in which the SMG and IPS contribute to the maintenance and selection of order information, while premotor and inferior frontal regions support articulatory rehearsal and the sequential organization of verbal material (Majerus et al. 2006; Papagno et al. 2017; Guidali et al. 2019; Majerus 2019). At the same time, evidence that positional information can be represented along multiple dimensions within overlapping fronto-parietal networks points to a distributed and flexible representational architecture (Attout et al. 2026).

Here, we tested whether different aspects of serial order are expressed as dissociable neural signatures. Participants memorized sequences presented left-to-right or right-to-left (Guida et al. 2020), thereby manipulating the spatial component of the encoding context while keeping retrieval cues centrally presented. Direction-sensitive decoding would indicate representations tied to the spatial configuration established during encoding, whereas decoding that generalizes across directions would indicate representations of ordinal position that are not bound to this configuration. Because these possibilities are not mutually exclusive, we hypothesized that spatially context-dependent codes would emerge in parietal regions involved in attentional orienting, particularly the IPS. More general ordinal representations were expected to involve medial temporal regions, including the hippocampus, as well as regions implicated in verbal serial order processing such as the supramarginal gyrus (SMG) (Papagno et al. 2017; Majerus 2019).

Using multivoxel pattern analysis (MVPA) and parametric modulation during retrieval, we tested for position-specific neural codes, their generalization across spatial encoding directions, and activity scaling with serial position. This approach allowed us to characterize neural signatures that either depend on the spatial encoding context or generalize beyond it, focusing on bilateral parietal cortex and hippocampus while also reporting whole-brain exploratory effects.

## Material and methods

### Participants

Thirty-five right-handed young adults (mean age = 23.34 years, range = 18–29; 24 females) were recruited from Ghent University and paid €35. All were right-handed, MRI-safe, and reported no neurological or psychological disorders. The study was approved by the UZ Gent Ethics Committee (approval number: B6702021000980), and all participants gave written informed consent. Six participants were excluded due to low in-scanner accuracy (*n* = 2), excessive head motion (*n* = 1), or technical problems (*n* = 3), resulting in a final sample of 29 participants.

### Experimental design and paradigm

We used an adapted version of the paradigm from van Dijck et al. (2013), combined with the spatial manipulation introduced by Guida et al. (2020). The experiment included a training session outside the scanner and an fMRI session. Both followed the same three-phase block structure: encoding, dot-detection retrieval, and order verification (Fig. 1). Critically, fMRI analyses focused on cue-evoked activity during the retrieval phase, when retrieval cues were presented centrally, allowing us to test whether the spatial organization established during encoding was reinstated during retrieval. During encoding, participants memorized four digits, selected from 1–9 excluding 5, presented sequentially either from left to right or from right to left. Digits subtended 0.45° of visual angle and were separated horizontally by 4.5°. Encoding direction was task-irrelevant. During retrieval, each centrally presented digit cue required participants to determine whether the digit belonged to the memorized sequence. The cue was followed by a white dot presented in one of two lateral rectangles. Participants responded with a right-hand key press only when the cue belonged to the sequence and withheld responses when it did not. The order-verification phase assessed explicit memory for serial order.

**Figure 1:**
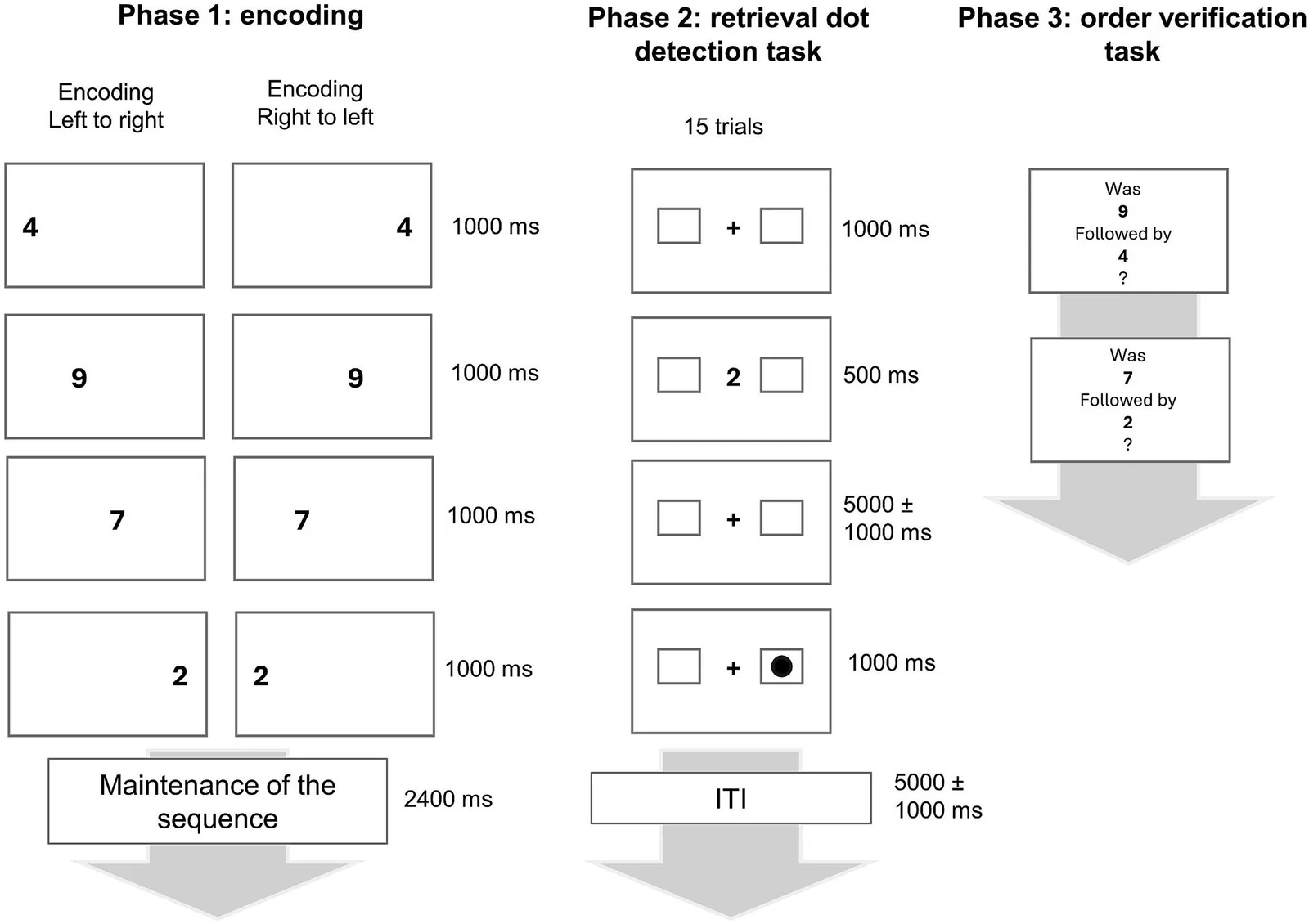
Experimental design. In Phase 1, participants encoded a digit sequence presented either from left to right or from right to left. In Phase 2, participants completed 15 dot-detection retrieval trials with centrally presented digit cues. In Phase 3, maintenance of the sequence order was assessed using two order-verification questions.

During *training*, participants completed 24 blocks, with encoding direction alternating every two blocks between left-to-right and right-to-left. In each block, four digits were presented sequentially for 1000 ms each, either from left to right or from right to left, separated horizontally by 4.5° of visual angle, followed by a 2500 ms rehearsal interval. Each block then included 20 retrieval trials. Each trial began with a central fixation cross (0.25° VA) flanked by two rectangles (1° × 0.67°, 3.2° eccentricity), followed by a digit cue presented for 300 ms, a short cue-target interval (mean = 250 ms, SD = 100 ms), and, on go trials, a white dot (0.5° × 0.5°) presented for 150 ms. Catch trials, in which sequence digits were not followed by a dot, discouraged anticipatory responses. Each block ended with three order-verification questions.

During *fMRI*, participants completed six runs, each containing four blocks, with two left-to-right and two right-to-left encoding blocks per run. The same three-phase structure was used, with timing adapted for event-related fMRI (Fig. 1). During encoding, digits were presented for 1000 ms each, either from left to right or from right to left, followed by a 2500 ms rehearsal interval. During retrieval, each trial began with a central fixation cross (1000 ms), flanked by two lateral rectangles, followed by a centrally displayed digit cue (500 ms), a jittered cue-target interval (mean = 5000 ms, SD = 500 ms), and, except on catch trials, a white dot presented for 200 ms in one of the two lateral rectangles. Participants had 1000 ms to respond on go trials, when the cue belonged to the memorized sequence, and to withhold responses on no-go trials, when the cue was not part of the sequence. Trials were separated by a jittered inter-trial interval (mean = 5000 ms, SD = 500 ms). Each block included 15 retrieval trials: 8 go trials, 5 no-go trials, and 2 catch trials in which sequence digits were not followed by a dot. Serial positions 1 and 4 were probed three times per block, whereas positions 2 and 3 were probed twice. Cue-target intervals and inter-trial intervals were jittered to allow event-related estimation of the BOLD response. Each block ended with two order-verification questions. Stimuli were presented using a Windows PC and projected onto a screen viewed through a mirror mounted on the head coil. Responses were collected using an MRI-compatible button box operated with the right index finger.

### Behavioral analysis

For the training phase, only retrieval trials from blocks with fully correct final order verification were included, ensuring proper order WM maintenance. For each participant and encoding direction, mean reaction times during the retrieval phase were submitted to a 4 (ordinal position: 1–4) × 2 (dot location: left vs. right) repeated-measures ANOVA. To assess spatial coding, we followed Fias (1996): for each serial position, we calculated dRTs as RT right dot minus RT left dot and regressed these values on serial position. Individual slope coefficients were tested against zero using one-sample t-tests. Negative slopes indicate left-to-right coding, whereas positive slopes indicate right-to-left coding.

The same analysis was applied to the fMRI session. Based on prior work (Cristoforetti et al. 2022), spatial-attention effects were expected to be attenuated in the scanner because of the longer cue-target interval used for event-related fMRI.

### Functional magnetic resonance imaging data acquisition

Imaging was conducted using a 3 T Siemens Prisma MRI scanner (Siemens Medical Systems, Erlangen, Germany) equipped with a 64-channel head coil. A high-resolution T1-weighted scan was acquired using an MPRAGE sequence (TR = 2250 ms, TE = 4.18 ms, TI = 900 ms, acquisition matrix = 256 × 256, FOV = 256 mm, flip angle = 9°, voxel size = 1 × 1 × 1 mm). Phase and magnitude field maps were acquired to correct for magnetic field inhomogeneities (TR = 520 ms, TE1 = 4.92 ms, TE2 = 7.38 ms, voxel size = 3 × 3 × 3 mm, 50 slices). Functional data were collected across six runs using a T2^*^-weighted gradient-echo EPI sequence (TR = 1730 ms, TE = 30 ms, flip angle = 66°, image matrix = 84 × 84, FOV = 210 mm, voxel size = 2.5 × 2.5 × 2.5 mm, 50 slices, slice acceleration factor = 2). Functional slices were oriented along the hippocampal axis, and 470 volumes were acquired per run. Each run consisted of four experimental blocks. Participants’ heads were stabilized using a vacuum cushion. Noise cancelation was applied during functional image acquisition (OptoACTIVE, Optoacoustics Ltd, Moshav Mazor, Israel).

### Functional magnetic resonance imaging data analysis

Before preprocessing, DICOM images were converted to NIfTI format using DCM2NII and organized according to the BIDS specification (Gorgolewski et al. 2016). Preprocessing was performed in SPM12 (v7487) running on MATLAB R2016b. Anatomical scans were segmented into gray matter, white matter, and cerebrospinal fluid, and forward deformation fields were extracted for normalizing functional data. Functional preprocessing included realignment and unwarping, slice-timing correction, coregistration to the structural T1 image, normalization to MNI space, and spatial smoothing with a 4 mm FWHM Gaussian kernel. Volumes with translations > 3 mm or rotations > 3° were excluded, and one participant was removed due to excessive motion.

Retrieval-related activity was modeled at digit-cue onset. For the MVPA model, separate regressors were specified for early and late items in each encoding direction and block, yielding eight regressors of interest per run. Middle serial positions, target-dot onsets, no-go trials, and six motion parameters were modeled as regressors of no interest. All events were modeled as zero-duration and convolved with the canonical HRF.

MVPA was performed on beta images corresponding to cue-evoked activity for early and late item retrieval. Within-direction decoding classified early versus late items separately within each encoding direction using a linear classifier (C = 1) (Schrouff et al. 2012) and leave-one-run-out cross-validation, training on five runs and testing on the remaining run. Cross-direction decoding trained classifiers on one encoding direction and tested them on the other, and vice versa, to assess whether serial-position-related patterns generalized across spatial encoding directions. As a control, we repeated cross-direction decoding using a leave-one-run-out scheme analogous to the within-direction decoding scheme, training on five runs from one encoding direction and testing on the remaining run from the opposite direction.

MVPA analyses used whole-brain searchlight decoding (10 mm radius; Decoding Toolbox; Hebart et al. 2015), restricted to a gray matter mask. Accuracy-minus-chance maps were submitted to second-level one-sample t-tests in SPM12. Statistical inference was first constrained using small-volume correction (SVC) within two a priori ROIs: bilateral parietal cortex (IPL, IPS, SPL) and bilateral hippocampus (Caspers et al. 2006, 2008; Choi et al. 2006; Scheperjans et al. 2008; Collin et al. 2015). Masks were defined using the Anatomy Toolbox (Eickhoff et al. 2007). Voxel-wise maps were thresholded at *P* < 0.001 uncorrected, with cluster-level FWE correction at *P* < 0.05 within each ROI. Exploratory whole-brain analyses used the same thresholds.

For the univariate parametric modulation analysis, a separate GLM tested whether BOLD activity scaled linearly with serial position during retrieval. Retrieval events were modeled at digit-cue onset with separate regressors for left-to-right and right-to-left encoding blocks, each including serial position (1–4) as a linear parametric modulator. Target-dot onsets, no-go trials, and six motion parameters were included as regressors of no interest. Given the regressor order of LR trial onset, LR parametric modulator, RL trial onset, and RL parametric modulator, direction-sensitive modulation was tested by comparing the two parametric modulators: [0 1 0–1] for stronger modulation in left-to-right than right-to-left encoding, and [0–1 0 1] for the reverse contrast. Direction-invariant modulation was tested by pooling the two parametric modulators [0 1 0 1]. The main effect of encoding direction, independent of serial position, was assessed with [1 0–1 0]. At the second level, contrast images were entered into group-level one-sample *t*-tests.

For the parametric analysis, SVC was applied within bilateral anterior hippocampal and bilateral parietal ROIs. The anterior hippocampal mask was defined according to Poppenk et al. (2013), based on evidence linking anterior hippocampus to generalizable relational coding and serial-position-related modulation (Poppenk et al. 2013; Brunec et al. 2018; Xu et al. 2026). To visualize activation across serial positions, we estimated an additional GLM in which positions 1–4 were modeled as separate conditions and extracted beta estimates from regions identified in the parametric modulation analysis.

## Results

### Behavioral results

We first analyzed behavioral performance during the training session to assess whether spatial coding of serial order differed by encoding direction. Analyses focused on correct go trials from successfully verified WM sequences (mean accuracy = 95.5%; go: 94%, no-go: 99%, catch: 98%).

In the left-to-right encoding condition, a 4 (ordinal position: 1–4) × 2 (dot location: left vs. right) repeated-measures ANOVA revealed a significant main effect of ordinal position [F(3,84) = 11.97, *P* < 0.001, ηp^2^ = .299], with RTs increasing across serial positions (361–392 ms). This was confirmed by a significant linear trend [F(1,28) = 35.86, *P* < 0.001, ηp^2^ = .562]. Crucially, there was an ordinal position × dot location interaction [F(3,84) = 2.72, *P* = 0.049, ηp^2^ = .089], consistent with a spatial shift from left to right across the sequence. A linear relationship was observed [F(1,28) = 5.62, *P* = 0.025, ηp^2^ = .167]. A regression of dRTs on position confirmed a significant negative slope [b = −0.007, t(28) = −2.37, *P* = 0.012], indicating faster responses to left-side targets for early items and to right-side targets for later items.

In the right-to-left encoding condition, the ANOVA revealed a significant main effect of dot location [F(1,28) = 5.02, *P* = 0.033, ηp^2^ = .152], indicating faster responses to right-side than left-side targets. The main effect of ordinal position showed a trend toward significance [F(3,84) = 2.45, *P* = 0.069, ηp^2^ = .081], with a significant linear trend across positions [F(1,28) = 5.31, *P* = 0.029]. However, no ordinal position × dot location interaction was observed [F(3,84) = 0.52, *P* = 0.667], and the dRT regression slope did not differ from zero [b = 0.003, t(28) = 0.93, *P* = 0.179], indicating no reliable spatial coding within the right-to-left condition. Nevertheless, a direct comparison of slopes between encoding directions revealed a significant difference [t(28) = 2.50, *P* = 0.019], indicating that spatial coding during retrieval was modulated by encoding direction (Fig. 2). No significant main effects or interactions were observed during the fMRI session (all *P*s > 0.10), consistent with the expected attenuation of spatial-attention effects at longer cue-target intervals (Cristoforetti et al. 2022).

**Figure 2:**
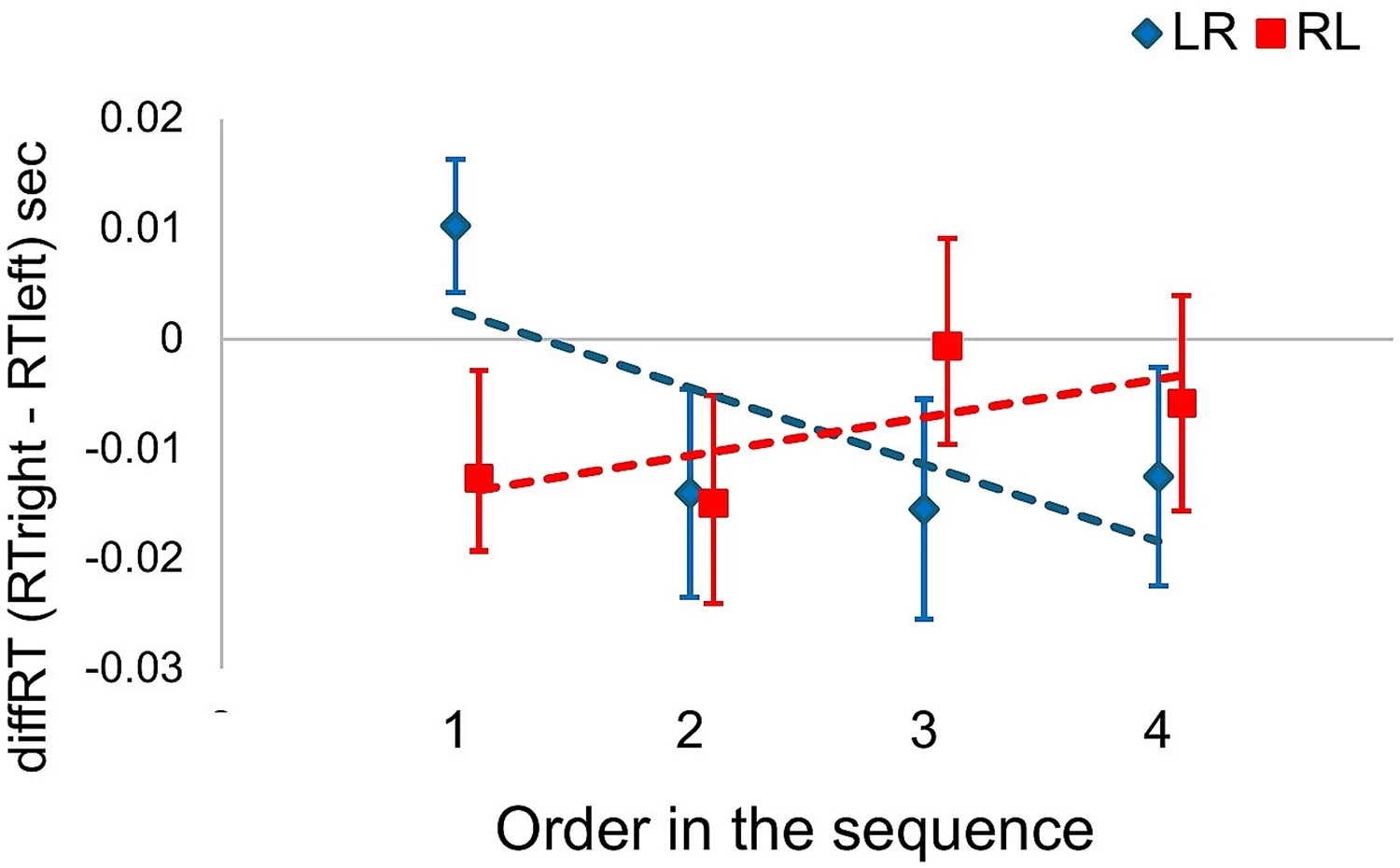
Differences between reaction times (RTs) to right- and left-side dots as a function of WM sequence position and encoding direction. The two encoding conditions, left-to-right and right-to-left, are shown separately. Each data point reflects the mean difference score across participants for a given ordinal position, and error bars represent the standard error.

### Functional magnetic resonance imaging results

#### Within-direction decoding

In a first set of MVPA analyses, we tested whether early versus late serial positions could be decoded separately within each encoding direction*.* MVPA analyses used whole-brain searchlight decoding (10 mm radius; Decoding Toolbox; Hebart et al. 2015), restricted to a gray matter mask. Accuracy-minus-chance maps were submitted to second-level one-sample t-tests. Statistical inference was first constrained using SVC within anatomically defined bilateral parietal and hippocampal ROIs, applying a voxel-wise threshold of *P* < 0.001 uncorrected and cluster-level FWE correction at *P* < 0.05.

For left-to-right encoding, searchlight decoding revealed significant above-chance classification within the parietal ROI in the left inferior parietal lobule, specifically PFm bordering the anterior IPS (MNI: −52, −50, 42; t(28) = 5.48; cluster size = 44 voxels; p FWE-corr = .001; Fig. 3A). No significant effects were found within the hippocampal ROI. At the whole-brain level, the same left inferior parietal cluster was significant (cluster size = 46 voxels; p FWE-corr = .005), together with an additional cluster in the left superior frontal lobule (MNI: −16, 58, 2; t(28) = 4.18; cluster size = 32 voxels; p FWE-corr = .042). These clusters are consistent with regions implicated in spatial attention, internal reorienting, and higher-order control processes, respectively (Rushworth et al. 2001; Dosenbach et al. 2006; Badre and D’Esposito 2007; Corbetta et al. 2008).

**Figure 3:**
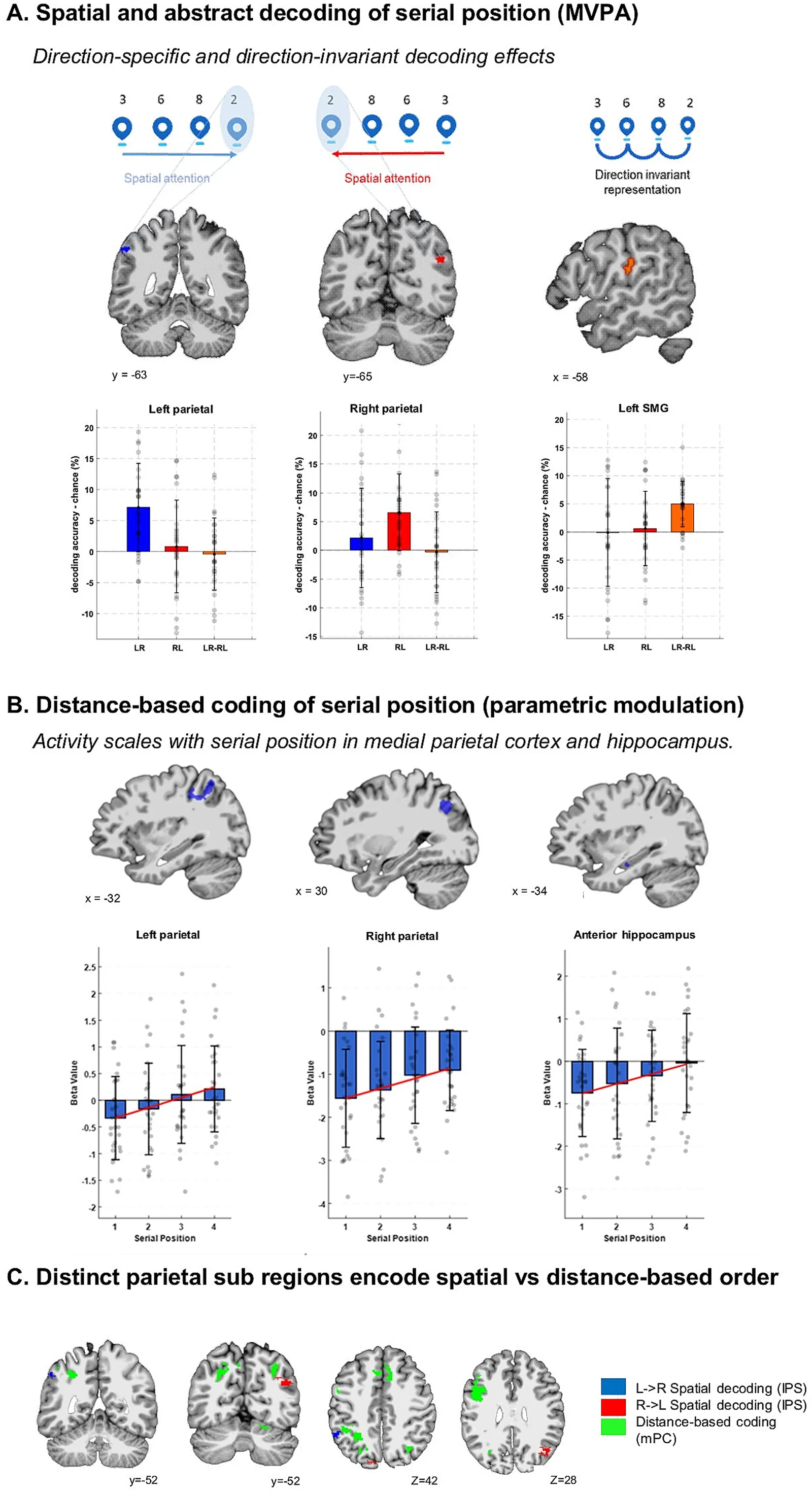
Direction-dependent and direction-invariant retrieval signatures of serial position in verbal working memory. **Panel A**. Neural decoding of serial position within and across spatial encoding contexts. For visualization, only clusters within the bilateral parietal ROI mask used for small-volume correction are displayed. Additional significant clusters outside this mask, such as precentral cortex, are reported in the text. Top row: brain maps illustrate significant clusters from decoding of serial position during retrieval. Left and middle panels show within-direction decoding for L → R and R → L encoding, respectively, revealing direction-specific position-related decoding in left and right parietal cortices. The right panel shows cross-direction decoding, with significant decoding in left SMG, indicating direction-invariant retrieval-related decoding of serial position. Bottom row: decoding accuracy minus chance, shown as mean ± SD, for each ROI. Left and right parietal cortices show above-chance decoding within their respective encoding directions but not across directions. In contrast, left SMG supports above-chance cross-direction decoding, consistent with direction-invariant retrieval-related signatures associated with serial position. **Panel B.** Parametric modulation of BOLD signal by serial position in parietal and hippocampal regions. Top row: brain maps show clusters exhibiting significant linear increases in activity across serial positions 1–4, identified via parametric modulation analysis during retrieval, using small-volume correction within the bilateral parietal and anterior hippocampal ROIs. The parietal clusters identified here lie in distinct subregions from those revealed by MVPA, namely lateral IPS versus medial PPC. Bottom row: mean beta values, shown as mean ± SD, as a function of serial position for each ROI. Bars represent average signal change at each position; superimposed lines indicate the best-fitting linear trend. Both parietal regions show monotonic increases in activation with serial position, consistent with a graded positional gradient during retrieval. A similar trend is observed in the left anterior hippocampus. **Panel C.** Distinct parietal subregions support spatial reinstatement and serial-position-related modulation. Overlays show direction-specific decoding localized to lateral IPS for L → R and R → L encoding, together with direction-invariant serial-position-related modulation in medial posterior parietal cortex. The lateral–medial separation of these clusters highlights a functional dissociation within parietal cortex: lateral IPS supports direction-dependent spatial reinstatement during retrieval, whereas PPC shows a positional gradient independently of encoding direction.

We next examined decoding accuracy for early versus late items from sequences encoded from right to left. As in the previous analysis, SVC revealed a significant cluster in the right inferior parietal cortex, corresponding to PGp within the angular gyrus and extending toward the IPS (MNI: 46, −66, 26; t(28) = 4.77; cluster size = 21 voxels; p FWE-corr = .041; Caspers et al. 2008; Fig. 3A). No significant decoding was observed within the hippocampal ROI. At the whole-brain level, significant clusters emerged in the left superior temporal gyrus (MNI: −58, −18, 8; t(28) = 4.93; cluster size = 40 voxels; p FWE-corr = .012), left fusiform gyrus (MNI: −38, −44, −20; t(28) = 4.79; cluster size = 35 voxels; p FWE-corr = .025), and right precentral gyrus (MNI: 48, 0, 36; t(28) = 4.68; cluster size = 37 voxels; p FWE-corr = .018). These whole-brain clusters are consistent with regions implicated in verbal/phonological, articulatory, and motor-related processes that may support retrieval (Majerus et al. 2006; Majerus 2019).

Bayesian one-sample t-tests confirmed extreme evidence for above-chance decoding in the left parietal cluster after left-to-right encoding (BF₁₀ = 4229) and in the right parietal cluster after right-to-left encoding (BF₁₀ = 3057). Hippocampal decoding showed moderate evidence for the null in the left-to-right condition (BF₀₁ = 7.87) and anecdotal evidence in the right-to-left condition (BF₀₁ = 2.4), indicating robust direction-specific decoding in parietal cortex but limited evidence for hippocampal decoding.

#### Cross-direction decoding

To test whether serial position representations generalized across spatial encoding directions, we conducted a cross-direction decoding analysis in which classifiers were trained on one encoding direction and tested on the other, and vice versa. SVC was applied within the same anatomically defined parietal and hippocampal ROIs as in the within-direction analyses. This analysis revealed a significant cluster in the left supramarginal gyrus (SMG; MNI: −62, −22, 20; t(28) = 4.45; cluster size = 23 voxels; p FWE-corr = .026; Fig. 3A). No significant decoding was observed within the hippocampal ROI.

At the whole-brain level, significant clusters were observed in the left SMG, at the boundary with the postcentral gyrus (MNI: −58, −22, 20; t(28) = 5.17; cluster size = 23 voxels; p FWE-corr = .002), and in the left precentral gyrus (MNI: −50, −6, 46; t(28) = 5.45; cluster size = 64 voxels; p FWE-corr < .001). These clusters are consistent with serial-order and motor-related retrieval processes (Majerus 2019).

As an additional control, we repeated the cross-direction decoding using a leave-one-run-out scheme analogous to the within-direction analysis, training on five runs from one direction and testing on the remaining run from the opposite direction. Under this reduced dataset, a significant cluster was observed in the left precentral gyrus at the whole-brain level (MNI: −50, −8, 48; cluster size = 134 voxels; p FWE-corr < .001). By contrast, decoding in the left SMG was no longer significant under SVC (MNI: −64, −20, 20; cluster size = 17 voxels; p FWE-corr = .088), suggesting that the SMG effect was more sensitive to dataset size and statistical power, whereas the precentral effect was more robust.

Bayesian one-sample t-tests showed moderate evidence for the null for within-direction SMG decoding (BF₀₁ = 5.2 for left-to-right; BF₀₁ = 3.3 for right-to-left), whereas cross-direction decoding showed extreme evidence for above-chance classification (BF₁₀ > 1000). This suggests that the SMG may preferentially carry direction-invariant retrieval-related information associated with serial position.

*Parametric modulation analysis*. Having identified regions whose multivariate activity distinguished between early and late serial positions, we next tested whether univariate activity scaled linearly with the serial position of the retrieved item. This analysis was motivated by serial scanning accounts of WM (Sternberg 1966; Marshuetz 2005) and by recent evidence that parietal and hippocampal activity can scale with relational distance in long-term memory tasks (Xu et al. 2026). We first tested whether serial-position-related modulation differed between left-to-right and right-to-left encoding, but found no significant clusters within the parietal or hippocampal ROIs or at the whole-brain level. Bayesian tests also favored the null hypothesis (BF₀₁ ≈ 2.3–7.5), indicating that serial-position-related BOLD scaling did not reliably differ between encoding directions.

We then examined direction-invariant parametric modulation. Within the bilateral parietal ROI, BOLD activity increased linearly with serial position in bilateral IPS. In the left hemisphere, the peak was located along the medial wall of the IPS, extending into superior and inferior parietal lobules (hIP3; MNI: −32, −56, 42; t(28) = 5.33; p FWE-corr < .001; Richter et al. 2019). In the right hemisphere, the peak was located in the lateral wall of posterior IPS (hIP5; MNI: 32, −66, 40; t(28) = 4.82; p FWE-corr = .001; Richter et al. 2019). Activation in both clusters increased monotonically across serial positions (Fig. 3B), consistent with a positional gradient during serial access. The parietal clusters identified by parametric modulation were located in distinct subregions from those revealed by MVPA, as illustrated in Fig. 3C.

We also observed serial-position-related modulation in the left anterior hippocampus (MNI: −34, −16, −18; t(28) = 4.06; cluster size = 5 voxels; p FWE-corr = .047).

At the whole-brain level, serial-position-related modulation was observed in bilateral supplementary motor area, a region commonly linked to temporal processing (left SMA: MNI: −6, 18, 48; t(28) = 6.10; cluster size = 144 voxels; p FWE-corr < .001; right SMA: MNI: 12, 18, 44; t(28) = 5.22; cluster size = 118 voxels; p FWE-corr = .001; Protopapa et al. 2019). Additional effects were observed in the left precentral sulcus (MNI: −56, 4, 20; t(28) = 7.42; cluster size = 634 voxels; p FWE-corr < .001), right inferior frontal gyrus (MNI: 42, 22, 4; t(28) = 5.03; cluster size = 112 voxels; p FWE-corr = .002), and insula (MNI: −30, 22, 4; t(28) = 5.83; cluster size = 338 voxels; p FWE-corr < .001), regions commonly associated with motor planning, sequential control, cognitive control, and attentional processes (Kastner et al. 1999; Corbetta and Shulman 2002; Majerus 2019; Fig. 4).

**Figure 4:**
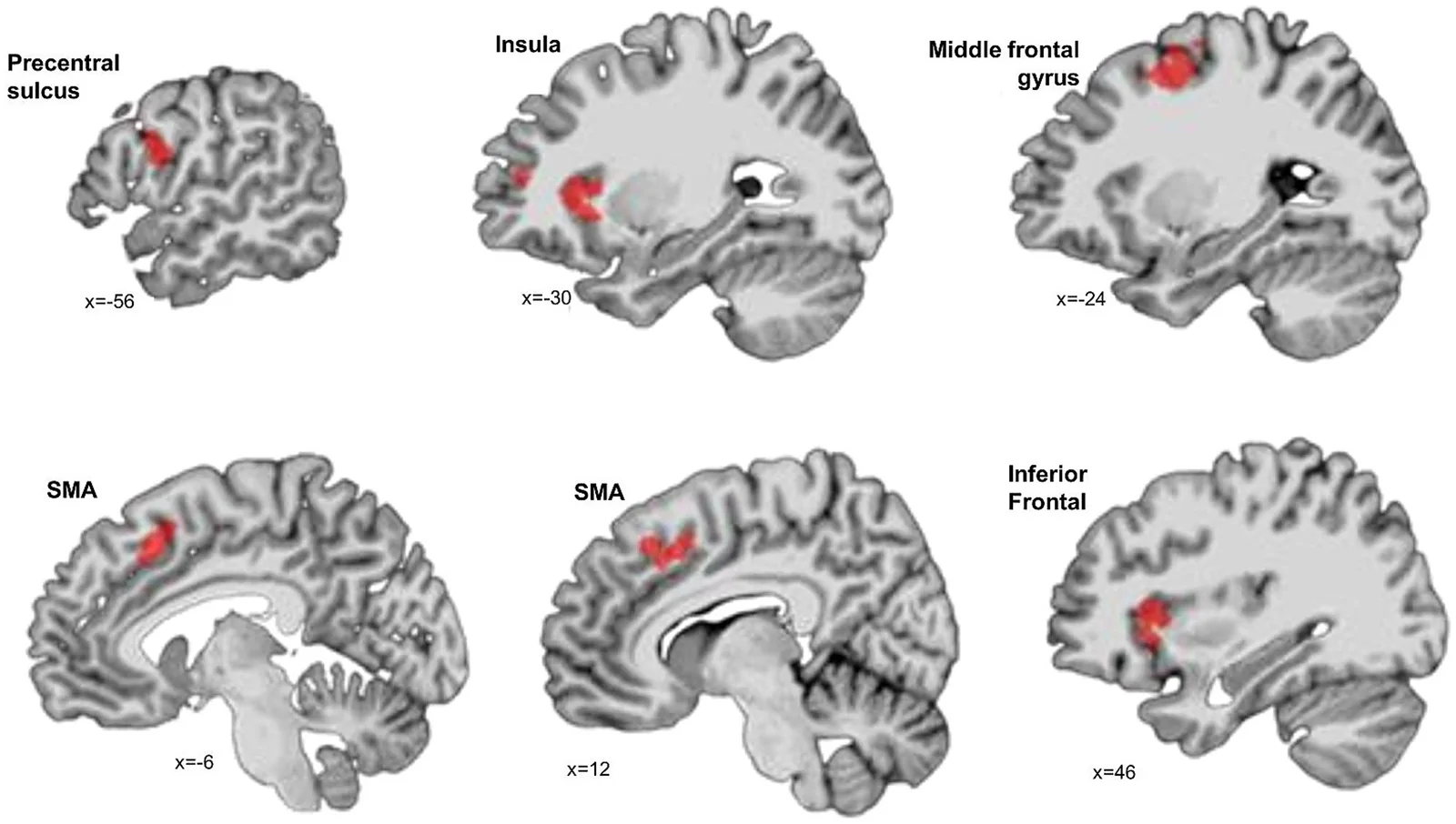
Whole-brain parametric modulation of BOLD signal by serial position across encoding directions (FWE-corrected).

## Discussion

In the present study, we investigated whether serial order retrieval in WM is supported by distinct representational components. By manipulating spatial encoding direction while keeping retrieval cues centrally presented, we dissociated neural signatures that preserve information about the spatial configuration of encoding from those that generalize beyond this structure. Our findings suggest that serial order is supported by a flexible, multidimensional system in which spatial organization constitutes one organizing principle alongside representations that generalize across spatial layouts.

Using MVPA together with parametric modulation of fMRI data, we characterized both the representational format and retrieval-related processing of serial order information. MVPA revealed a dissociation between direction-specific effects in lateral parietal cortex, linked to the spatial organization established during encoding, and spatially invariant effects in supramarginal and premotor regions, suggesting serial-position information that generalized across encoding directions. Parametric modulation provided a complementary index of retrieval-related computational demands, revealing graded activity consistent with sequential access.

Serial position could be decoded in lateral parietal regions in a direction-specific manner, with left-lateralized decoding after left-to-right encoding and right-lateralized decoding after right-to-left encoding. Because spatial information was available only during encoding and all retrieval cues were presented centrally, these effects are unlikely to reflect perceptual processing of current stimulus location. At the same time, because serial order and spatial position were systematically mapped onto each other during encoding, they should not be interpreted as reflecting serial position independently of the spatial layout. If parietal activity reflected a purely temporal representation of serial order, decoding should have generalized across encoding directions, given that temporal order was identical across conditions. Instead, the observed direction-specific patterns suggest that retrieval reinstates an internally constructed spatial organization shaped by the encoding context, rather than a veridical copy of the encoding layout or purely stimulus-driven spatial features. This interpretation is consistent with behavioral evidence that verbal order can be reconstructed by relying on the sequence of spatial locations associated with successive items, suggesting that remembered sequence structure can incorporate both ordinal and spatial information (Paron et al. 2026). The observed hemispheric pattern is consistent with the contralateral organization of the dorsal attention network (Corbetta and Shulman 2002; Nobre et al. 2004), suggesting that direction-specific parietal effects may reflect covert shifts of spatial attention along an internally constructed spatial axis (van Dijck et al. 2013). Behavioral evidence for this interpretation was asymmetric: left-to-right encoding produced a significant rightward attentional bias, whereas right-to-left encoding produced only a numerically opposite, non-significant trend. The right-parietal effect should therefore be regarded as consistent with, rather than direct evidence for, a reversal of covert spatial attention. This asymmetry may reflect the influence of participants’ habitual left-to-right reading direction, which culturally determines spatialization in WM (Guida et al. 2018; Rasoulzadeh et al. 2024), and, more speculatively, the within-participant alternation between habitual and reversed layouts (Guida et al. 2020). Nevertheless, the significant difference between slopes indicates that encoding direction modulated spatial coding.

In contrast, cross-direction decoding revealed serial-position information in the left supramarginal gyrus (SMG) that generalized across spatial layouts. Because this analysis collapses across encoding directions, these effects cannot be explained by spatial position differences between early and late items, but instead reflect serial position information not tied to the spatial organization established during encoding. Given the role of SMG in domain-general serial order processing and in integrating item identity with positional information (Papagno et al. 2017; Majerus 2019), this effect may reflect a direction-agnostic ordinal code. More broadly, intracranial evidence suggests that the SMG functions as a parietal node supporting attentional control over memory across verbal and spatial episodic-memory tasks, with behaviorally relevant directed interactions from SMG to hippocampus (Das and Menon 2024b). Within-direction decoding in the SMG did not reach significance, but these analyses involved fewer trials and reduced statistical sensitivity. The leave-one-run-out control further showed that the SMG effect was sensitive to statistical power, suggesting that the absence of robust within-direction decoding should be interpreted cautiously.

We also identified a significant cluster in the left precentral gyrus showing spatially invariant decoding of serial position. This region lies near the hand area of motor and premotor cortex, and both MVPA and parametric modulation analyses revealed converging effects. Given its proximity to dorsal premotor regions and the established involvement of premotor cortex in response preparation, action sequencing, and the motoric support of working-memory rehearsal, this pattern may reflect graded motor-based processes engaged during serial retrieval (Marvel et al. 2019). Accessing later serial positions may require greater internal rehearsal or increased preparatory engagement, leading to systematic differences in motor-related BOLD responses. This interpretation aligns with evidence from visuo-spatial and motor sequence tasks showing that premotor activity reflects prospective planning of sequential actions (Langner et al. 2014).

Unexpectedly, hippocampal decoding of serial position was absent. This null result may reflect either limited sensitivity to a weak or unstable hippocampal signal, or a genuine absence of an explicit representation of absolute serial position under the present task conditions. This contrasts with our previous findings under fixed encoding conditions, in which centrally presented sequences yielded hippocampal decoding (Cristoforetti et al. 2022). We expected hippocampal involvement based on prior studies linking this region to viewpoint-invariant relational coding across spatial and non-spatial domains (Milivojevic and Doeller 2013; Constantinescu et al. 2016; Bottini and Doeller 2020). Frequent alternation between left-to-right and right-to-left encoding may have disrupted the emergence of stable hippocampal signals, thereby hindering the formation of representations that generalize across spatial layouts. Under more consistent conditions, hippocampal and parietal representations may align to support transformations between reference frames. By contrast, when integration across inconsistent spatial encoding layouts is required, hippocampal representations may need longer consolidation time (Stachenfeld et al. 2017). In environments with directional ambiguity or frequent changes in spatial layout at encoding, such integration may be hindered. Supporting this view, rodent studies have shown that hippocampal place cells, which are omnidirectional in 2D spaces, become direction-specific in constrained 1D environments, such as tunnels (Derdikman et al. 2009; Geva-Sagiv et al. 2015). Alternatively, hippocampal involvement may have been unnecessary because spatial structure was already externally provided by the physical layout of the sequence, allowing parietal areas to support order through spatially anchored visuospatial codes. These interpretations highlight a flexible division of labor between parietal and hippocampal systems, depending on contextual demands and representational requirements.

Although no direction-independent position codes were observed in the hippocampus, the left anterior hippocampus showed direction-invariant parametric modulation, with BOLD activity increasing linearly with serial position. This dissociation suggests that hippocampal involvement may be expressed as a graded relational signal rather than as a stable local code for absolute serial position. Consistent with this interpretation, intracranial evidence from verbal episodic memory shows that hippocampal activity tracks distances between items in semantic and temporal representational spaces (Solomon et al. 2019b). Similar parametric modulation was observed in bilateral parietal areas, located more medially than the lateral parietal regions showing direction-specific position decoding, as well as in the supplementary motor area. Together, these findings suggest that these regions express a positional gradient, potentially reflecting a serial scanning strategy during retrieval. This interpretation aligns with classic scanning models of verbal WM and with serial-position-related effects reported in previous tasks (van Dijck et al. 2013; Attout et al. 2014; De Belder et al. 2015; Guida et al. 2020). Importantly, this positional gradient was invariant across encoding directions, indicating that it reflects a context-independent component of serial order retrieval that generalizes across spatial layouts.

Converging evidence for serial-position-related modulation in posterior parietal cortex (PPC) and anterior hippocampus has been reported by Xu et al. (2026). In their task, participants learned a temporal sequence of daily events and judged whether probe events occurred in the same or different parts of the day. Neural activity in both hippocampus and parietal cortex scaled with temporal distance between events. Importantly, Xu et al. included a perspective manipulation and showed that hippocampal effects were perspective-invariant, whereas parietal effects varied across perspectives, but not temporal direction. Although our design does not allow a direct test of temporal perspective, the present findings extend this work by showing a positional gradient during serial access in WM.

The serial-position-related activity observed across SMA, precentral sulcus, insula, middle frontal gyrus, and inferior frontal cortex may reflect broader control processes supporting the temporal organization of serial access, rather than a representation of serial position per se. These regions overlap with systems implicated in motor timing, task maintenance, action planning, and action-oriented control, including the cingulo-opercular/action-mode network (Nani et al. 2019; Protopapa et al. 2019; Dosenbach et al. 2025). In this context, the parametric increase with serial position may index increasing demands associated with maintaining the task set, preparing responses, and progressively unfolding the sequence in WM. Consistent with this interpretation, reaction times increased with serial position regardless of encoding direction, suggesting greater access or control demands for later positions.

Together, the coexistence of direction-dependent spatial reinstatement, spatially invariant position-related representations, and graded serial-position effects indicates that serial-order retrieval relies on multiple, dissociable components. These regional signatures should nevertheless be understood within a broader systems-level architecture. Recent evidence from sequential working-memory tasks indicates that the maintenance and active reorganization of sequence information recruit distributed frontoparietal representations and demand-dependent network reconfiguration, involving parietal, prefrontal, insular, premotor, and supplementary motor regions (Wang 2026). Complementary intracranial studies further indicate that verbal and spatial memory involve dynamic coordination between the hippocampus and parietal, frontal, and other medial temporal regions (Solomon et al. 2019a; Herweg et al. 2020; Das et al. 2022; Das and Menon 2022, 2024a, 2024b).

Because centrally presented sequences were not included within the same design, the present study cannot determine whether the representations observed under explicit spatial layouts share a common neural code with those formed under central presentation. Future studies directly comparing central, left-to-right, and right-to-left encoding within participants could address whether serial-position representations generalize across these different encoding formats. Another line for future research could combine representational decoding with hypothesis-driven connectivity analyses to determine how spatially specific and spatially invariant serial-order representations are coordinated within these distributed networks.

In conclusion the MVPA results suggest distinct representational formats: spatially grounded representations in parietal cortex preserve information about the organization established during encoding, whereas spatially invariant representations in supramarginal and premotor regions support access to serial position beyond this spatial structure. In contrast, the parametric effects are better interpreted as processing demands that scale with serial position, with posterior parietal cortex, hippocampus, SMA, insula, and frontal regions indexing a positional gradient associated with progressive access or unfolding of the sequence. These findings suggest a functional division between representational and computational components: serial order is represented in both spatially specific and spatially invariant formats, while access to these representations may involve graded control or sequencing processes that increase for later positions. Serial order in WM therefore does not rely on a single code, but emerges from the interaction between how information is represented and how it is accessed.
